# Thalidomide attenuates the hyporesponsiveness of isolated atria to chronotropic stimulation in BDL rats: The involvement of TNF-α, IL-6 inhibition, and SOCS1 activation

**DOI:** 10.22038/ijbms.2019.32256.7742

**Published:** 2019-11

**Authors:** Ali Hosseini-Chegeni, Farahnaz Jazaeri, Aliakbar Yousefi-Ahmadipour, Mansour Heidari, Alireza Abdollahie, Ahmad Reza Dehpour

**Affiliations:** 1 Department of Pharmacology, School of Medicine, Tehran University of Medical Sciences, Tehran, Iran; 2 Department of Laboratory of Sciences, Faculty of Paramedical Sciences, Rafsanjan University of Medical Sciences, Rafsanjan, Iran; 3 Department of Medical Genetics, Tehran University of Medical Sciences, Tehran, Iran; 4 Department of Pathology, School of Medicine, Imam Hospital Complex, Tehran University of Medical Sciences, Tehran, Iran; 5 Experimental Medicine Research Center, Tehran University of Medical Sciences, Tehran, Iran

**Keywords:** Cirrhotic cardiomyopathy, IL-6, SOCS1, Thalidomide, TNF-α

## Abstract

**Objective(s):**

Cirrhotic cardiomyopathy is a complication of uncured cirrhosis which is associated with hyporesponsiveness of the heart to sympathetic stimulation. The enhancement of portal pressure, nitric oxide (NO) level, pro-inflammatory mediators and down-regulation of Suppressor of Cytokine Signaling 1 (SOCS1) are involved in this situations. The present study seeks to examine the beneficial effect of thalidomide on cirrhotic cardiomyopathy.

**Materials and Methods:**

The male rats were grouped as: Sham/saline, Sham/Thalidomide, Bile Duct Ligation (BDL)/saline and BDL/Thalidomide. BDL model of cirrhosis was used. In the treatment groups, thalidomide (200 mg/kg/day) was administrated by intragastrial gavage for 28 consecutive days, the chronotropic response was assessed in isolated atria by isoproterenol stimulation. Serum levels of NO, IL-6 and TNF-α hepatic level were evaluated. The intrasplenic pulp pressure (ISPP) as the portal pressure and histopathologic assessment were assessed. Real time RT-PCR was used for the evaluation of SOCS1 gene expression.

**Results:**

Our results showed that thalidomide administration could significantly increase the atrial chronotropic response in BDL animals. The increased level of portal pressure decreased by thalidomide in BDL animals. Thalidomide could ameliorate the histopathological conditions of BDL rats. Furthermore, the chronic treatment by this drug diminished the elevated levels of NO, TNF-α and IL-6 in BDL animals. On the other hand, hepatic SOCS1 expression was up-regulated by thalidomide treatment in this group.

**Conclusion:**

Thalidomide improves the chronotropic hyporesponsiveness of isolated atria in BDL. This effect is probably mediated by the inhibiting NO, TNF-α and IL-6 production, reducing portal pressure and increasing the expression of SOCS1.

## Introduction

Liver cirrhosis is defined by the end-stage of extended fibrosis, regenerative nodules and widespread inflammation (1). Cirrhotic cardiomyopathy is a cirrhosis complication which is characterized by impaired inotropic and chronotropic responsiveness to sympathetic stimulation (2). One of the main causes of cirrhotic cardiomyopathy is the hyperdynamic circulatory syndrome (HC) which is associated with portal hypertension (PTH), systemic and splanchnic NO overproduction. PTH in cirrhosis is caused by increased intrahepatic resistance (IHR). In response to this event, the over release of splanchnic NO and overall hypotension occur. To compensate for this condition, cardiac chronotropic increases that in turn induces the decline of heart function and myocardium contraction in the long term (3). On the other hand, the intensification and progression of liver fibrosis is related to the enhancement of TNF-α and IL-6 levels in the liver (4, 5). Furthermore, TNF-α plays a pivotal role in increasing NO in cirrhosis (6) and also in NO overproduction (7). Suppressor of Cytokine Signaling 1 (SOCS1) is a suppressor of cytokine signaling that has a role in the modulation of cytokine-mediated immune responses (8). Several studies have indicated that during hepatic chronic diseases and cirrhosis, the expression of SOCS1 decreases (9-11) and this expression diminishes along with cirrhosis progression (11). Isoproterenol is a potent agonist of β-adrenoceptors that has positive inotropic and chronotropic effects on the myocardium. These effects of β1-adrenoceptors are applied through G-protein coupled receptor and the rise of cAMP (12). Previous studies have exhibited that responsiveness to isoproterenol reduces in cirrhosis (3). Thalidomide (N-α-phthalimidoglutarimid) is an old drug with immunomodulatory and anti-inflammatory effects that are used in the treatment of diseases with TNF-α involvement (13). One of the main effects of thalidomide in cirrhosis is mediated by the reduction of hepatic fibro- inflammation. This effect is probably exerted by the inhibition of collagen deposition and also anti-inflammatory effects that are mediated by the prevention of TNF-α and IL-6 production (14). Chong *et al*. reported that thalidomide could decrease liver fibrosis by the inhibition of TNF-α, and activate hepatic stellate cells in dimethylnitrosamine-intoxicated rats (14). In another study, Yang *et al*. noted that thalidomide could decrease portal vein pressure (PVP) through the reduction of TNF-α level in BDL rats (15). Regarding all of the above-mentioned evidence and given the insufficient number of studies concerning the effects of thalidomide in cirrhotic cardiac disorders, we decided to examine the possible mechanism of action as well as the effect of thalidomide on the chronotropic hyporesponsiveness of isolated atria from BDL rat to β-adrenergic stimulation.

## Materials and Methods

All materials were purchased from Sigma (Pool, UK), unless indicated in the text, otherwise. All animal procedures were in agreement with “Guide for the Care and Use of Laboratory Animals” (NIH US publication No 85-23, revised1985) recommendations.

***Animals***

Male Wistar albino rats (body weight around 250–280 g) were used for this study. Animals were obtained from the Department of Pharmacology (Tehran University of Medical Sciences). Animals were housed in a normal temperature (around 22 ^°^C), controlled humidity and light-dark cycle of 12 hr. Food and tap water provided *ad libitum* too.

The animals were randomly distributed into four groups, namely; sham-operated +saline group (sham/Saline), bile duct ligated cirrhotic+ saline group (BDL/Saline), sham-operated + Thalidomide group (sham/Thl), bile duct ligated+ Thalidomide (BDL/Thl). Thalidomide was administrated at dose 200 mg/kg/day by intragastrically gavage route for 28 days, beginning from the day of surgery. Each group consisted of 7 rats. All studies related to animals were done in agreement with the standards guidelines of animal care and the Council Directive of Laboratory Animals, Tehran University of Medical Sciences, Iran. 

***Cirrhosis induction***

For the general anaesthesia, ketamine 100 mg/kg and xylazine 8 mg/kg used intraperitoneally. Cirrhosis was induced via double ligation-section of the common bile duct, consistent with the previously described method (16) . The sham procedure involved a similar operation but without ligation or cutting of the bile duct. All animals were anesthetized and sacrificed at day 28 post operation when cirrhosis had established.

***Preparations of isolated atria***

For the study of cardiac chronotropic response to adrenergic stimulation, isolated beating left and right atria were used. Hence, after general anaesthesia by ketamine 100 mg/kg and xylazine 0.05 mg/kg, intraperitoneally, the rat atria were isolated in cold oxygenated physiological salt solution; it has been suspended in a 20 ml organ bath chamber under isometric tension of 1 g force. The composition of physiological solution in millimolars was as follows: NaCl, 112; KCl, 5; CaCl_2_, 1.8; MgCl_2_, 1; NaH_2_PO_4_, 0.5; KH_2_PO_4_, 0.5; NaHCO_3_, 25; glucose, 10; and EDTA, 0.004 and pH 7.35-7.45 that aerated with 95% O_2_ and 5% CO_2_.The right atrium was used for recording the spontaneous atrial beating, that was stimulated by cumulative concentrations of isoproterenol from 10-10 to 10-6mol/l. Tensions caused by atria beating, converted to electrical signals by Power Lab system (ADInstruments Pty Ltd. Australia). Recording, analysis, and display of electronic data, did by LabChart v8.1.11 software (ADInstruments Pty Ltd).

***Assessment of plasma nitrite/nitrate concentrations:***

Plasma nitrate and nitrite levels were measured as indicators of NO production The measurements were performed according to the method described previously (17). Plasma samples were deproteinized by centrifugation through a 30-kDa molecular weight filter (Centricon Millipore) at 14,000 rpm, for 1.5–3 hr at 4 °C. After filling the plate with samples (100 μl), adding of a saturated solution of VCl3 (100 μl) to each well was quickly followed by addition of the Griess reagents (50 μl each). Sulfanilamide and naphthylethylenediamine dihydrochloride were used for the preparation of Griess reagents. The plate was incubated at 37 ^°^C for 30 min and then absorbance at 540 nm was calculated using a standard plate reader (800™ TS Absorbance Reader, BioTek Instruments). Fresh standard solutions of nitrate were included in each test.

***Pathological examination***

Hepatic and serum level of TNF-α, and hepatic IL-6, levels were quantified with ELISA kits following the manufacturer’s directions (Quantikine Elisa) Hepatic amount of TNF-α was calculated per 1 g of wet tissue in 5 ml of phosphate buffer solution (PBS) and expressed as pg/g of liver tissue. The minimum detectable concentration of TNF-α is 1 pg/ml.

***Measurements of IL-6, TNF-*** ***α***

Hepatic and serum level of TNF-α, and hepatic IL-6, were quantified with ELISA kits following the manufacturer’s directions hepatic amount of TNF-α was calculated per 1 g of wet tissue in 5 ml of phosphate buffer solution (PBS) and expressed as pg/g of liver tissue. The minimum detectable concentration of TNF-α is 1 pg/ml.

***Assessment of intrasplenic pulp pressure (ISPP)***

As an index of portal vein pressure, ISPP was evaluated according to previously described techniques (18). Concisely, under general anesthesia (ketamine 100 mg/kg and xylazine 8 mg/kg IP), the spleen was exposed after the abdominal cavity opening; then a 20 gauge needle insert into the splenic parenchyma and intrasplenic pressure was evaluated (18). The needle was related to a pressure transducer that was attached to a Power Lab system (ADInstruments Pty Ltd.Australia). The external zero reference point was placed at the middle part of animal. The pressure reading was accepted when a stable recording was attained. Splenic pulp pressure calibration was performed using an upright tube filled with saline solution and is expressed in mm H_2_O.

***Measurement of SOCS1 expression***

Rat liver samples were removed and quickly dipped in liquid nitrogen. Total RNA was isolated using RNeasy Fibrous Tissue Mini Kit (QIAGEN) in accordance to manufacturer’s guidelines. Genomic DNA was removed with DNase (Promega). First strand cDNA was created with deoxyribonuclease (DNase)-treated RNA, random hexamer primer (p (dN) 6) and ribonuclease-free water, and then warmed at 70 ^°^C for 5 min and placed on ice. Ribonuclease inhibitor, reverse transcriptase, and deoxynucleoside triphosphates were added and incubated at 42 °C for 1 hr. The primer sequences for the genes and internal standard were as follows (19):Rat SOCS1 (product size: 160): forward 5´-AGCTGTGTCGCCAGCGCATC-3´; SOCS1: reverse 5´-CAGAAGTGGGAGGCATCTCA-3´(20); Rat β-actin, forward: 5’-TCCTGGGTATGGAATCCTG-3’’, reverse: 5’- CTTCTGCATCCTGTCAGCAA-3’( product size: 190 bp).For real-time PCR we used of 1.0 µl of cDNA template, 100 nM each of forward and reverse oligonucleotide primers, and 10.0 µl of optimized PCR Mastermix in a total reaction volume of 20.0 µl, with a Rotor-Gene QRT-PCR cycler, followed by 38 cycles of the annealing step (45 s at 60 ^°^C) and an extension–elongation step (1 min at 72 ^°^C). For analyzing the data, we used the competitive critical threshold (ΔΔct) method, in which the SOCS1 RNA was adjusted to β-Actin.

***Statistical analysis***

Statistical analyses were carried out using GraphPad Prism 5.0 software (GraphPad Inc., SanDiego, CA, USA). The results are presented as mean±SEM. One-way ANOVA along with Bonferroni post-test was performed for multiple comparison where appropriate. Moreover, differences between non-normally distributed variables were examined by Kruskal–Wallis and Dunn’s test *post hoc*. *P*-values less than 0.05 were considered statistically significant.

## Results

**Figure 1:**
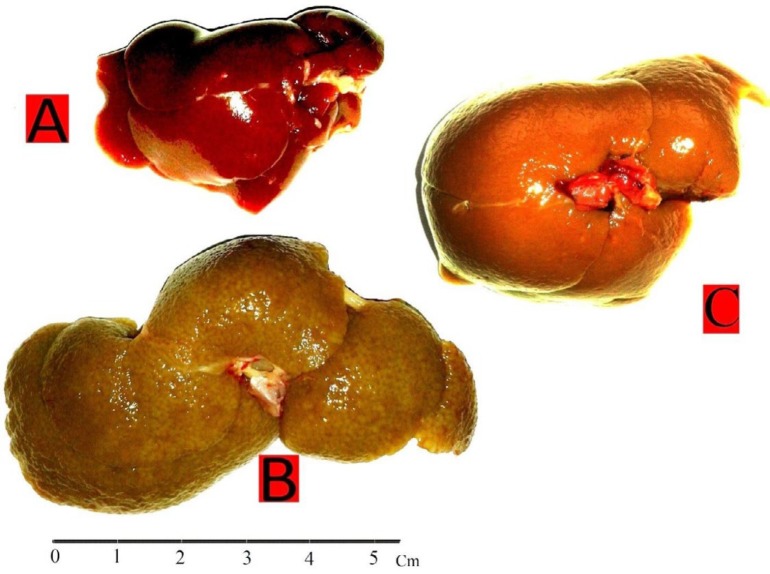
Macroscopic view of the rat liver: Control group (A); BDL group (B) with hepatomegaly, yellow pigmentation of bilirubin, macronodulation, grainy & pale surface; BDL/Thl group (C) improvement of cirrhotic conditions than BDL group

BDL: Bile Duct Ligated; Thl: Thalidomide

BDL rats shown signs of biliary cirrhosis such as jaundice: dark urine, steatorrhea and ascites from the third post-surgery. In BDL group the stiffness and liver size was more than control group. It also had a nodular appearance with pale colour (dark cream) in the BDL group compared to the control group (Figure 1). Therefore, we visually inspected the cirrhotic appearance of the liver in all experimental animals to conﬁrm the progression of cirrhosis.

***In vitro*** ***study***

**Figure 2:**
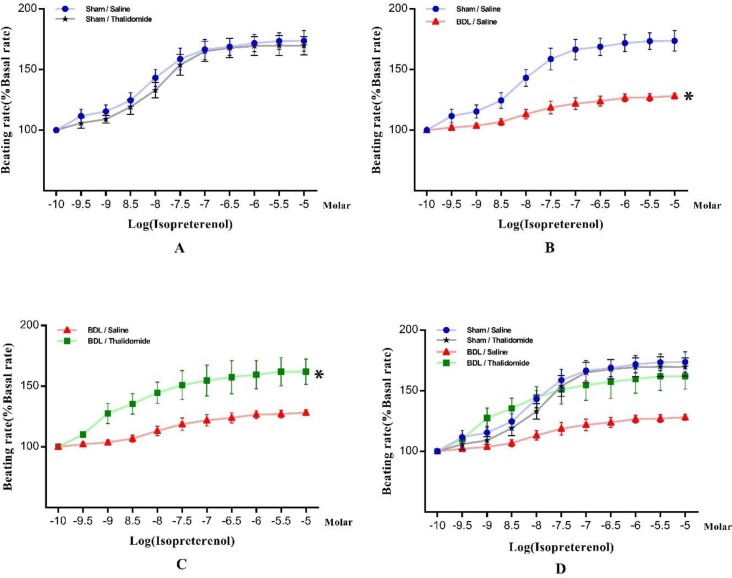
Chronotropic concentration-dependent responsiveness to isoproterenol stimulation that were obtained on spontaneous beating isolated, after 28 day, in all study groups (A), Sham- operated in comparison with Sham-operated treated with thalidomide (B), bile duct ligated than Sham-operated (C) bile duct ligated treated with thalidomide versus bile duct ligated. (D) All above mentioned groups. Data are expressed as % increase in basal beating rate and shown as mean±SEM, *P<0.05, (One-way ANOVA, bonferroni post-test) (n=7)

In this part of the experiments, we aimed to measure the rate of atrium responsiveness in the study groups to isoproterenol stimulant. Therefore, normalized graph of heart rate than increasing isoproterenol and ANOVA one-way test were used for comparison between study groups. In more details, our data showed that thalidomide administration has no effect in sham group on chronotropic response (Figure 2A). besides, chronotropic responses to isoproterenol were impaired signiﬁcantly in isolated atria following chronic bile duct ligation (FBDL vs sham =1.083,* P*<0.05, Figure 2B). However, the maximum response (R_max)_ to isoproterenol was lower in BDL/ Saline group in comparison with Sham/Saline group but this difference was not significance (Table 1). Furthermore, there was no signiﬁcant difference in isoproterenol EC_50_ between those groups (Data not be shown). Thalidomide could increase chronotropic responses to isoproterenol in BDL rats, signiﬁcantly (FBDL vs _BDL/ Thl_ = 2.041, *P*<0.05, Figure 2C). Although R_max_ was noticeably increased after thalidomide treatment in BDL/ Thalidomide rats (Table 1) it’s not signiﬁcant, EC_50_ of isoproterenol did not show a signiﬁcant difference between animals in BDL/ Saline than BDL/ Thalidomide groups (log EC_50_ = -7.550 ±0.23 and -8.450± 0.27, respectively).

***Intrasplenic pulp pressure***

**Figure 3:**
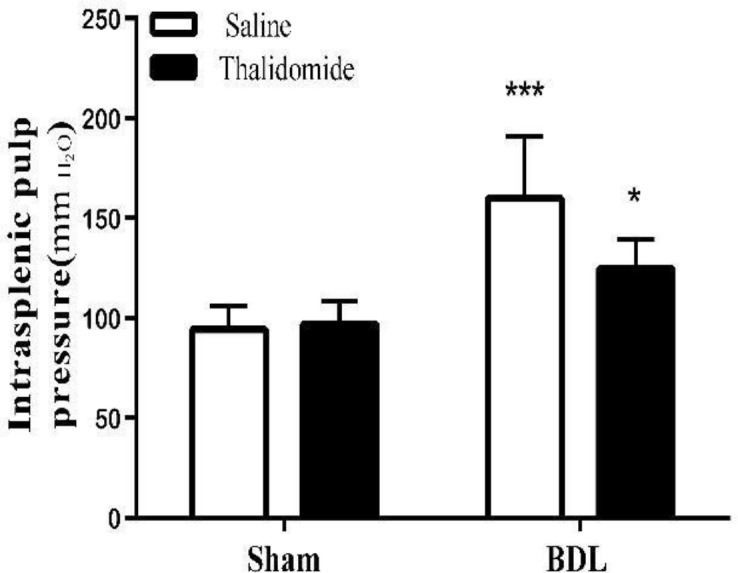
Increasing intrasplenic pulp pressure in sham- operated than bile duct ligated rats (***P<0.001) and its diminishing with thalidomide treatment (*P<0.05), mean±SEM. (One- way ANOVA, bonferroni post-test) (n=7)

Intrasplenic pulp pressure (ISPP) was measured and used in place of a portal venous pressure (PVP) index. In BDL/ Saline rats as shown in Figure 3, PVP increased signiﬁcantly 4 weeks after bile duct ligation (*P*<0.0001). Our data has shown that thalidomide can decrease PVP in BDL rats significantly (*P*<0. 05).

***Plasma nitrite/nitrate concentrations***

Assessment of serum nitrite/nitrate concentration used to NO systemic index; therefore, NO was significantly higher in the BDL/ Saline than Sham/ Saline group (*P*<0.001). Furthermore, it lessened in BDL/ Saline group in comparison with the BDL/ Thalidomide group significantly (Table 2).

***TNF-*** ***α*** ***,*** ***I*** ***L-6 levels***

Assessment of TNF-α and IL-6 levels in the study group by ELISA method has shown that chronic bile duct ligation can increase noted cytokines in liver significantly (PTNF-α <0.001, PIL-6 <0.01). Additionally, hepatic levels of these cytokines, in treated BDL rats with thalidomide, decreased significantly (*P*<0.05). Also TNF-α serum levels increased in BDL rats compared with sham-operated animals; moreover, TNF-α levels were signiﬁcantly decreased in BDL/ Thalidomide rat livers (Table 2).

***Quantitative measurement of hepatic SOCS1mRNA expression***

**Figure 4:**
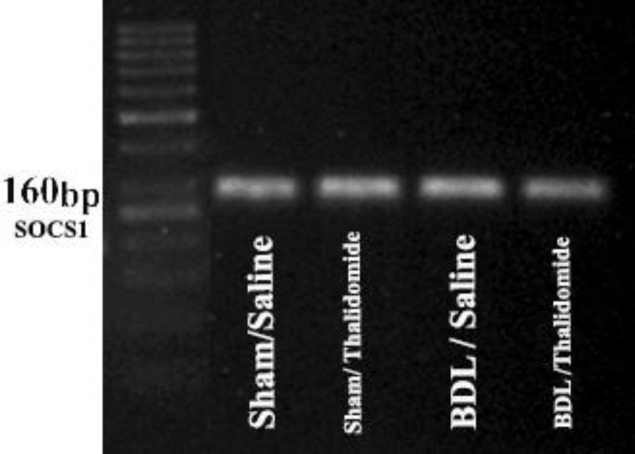
The expression of SOCS1 c-DNA obtained from RT-PCR in livers of study groups that appear in single band (n=7)

**Figure 5:**
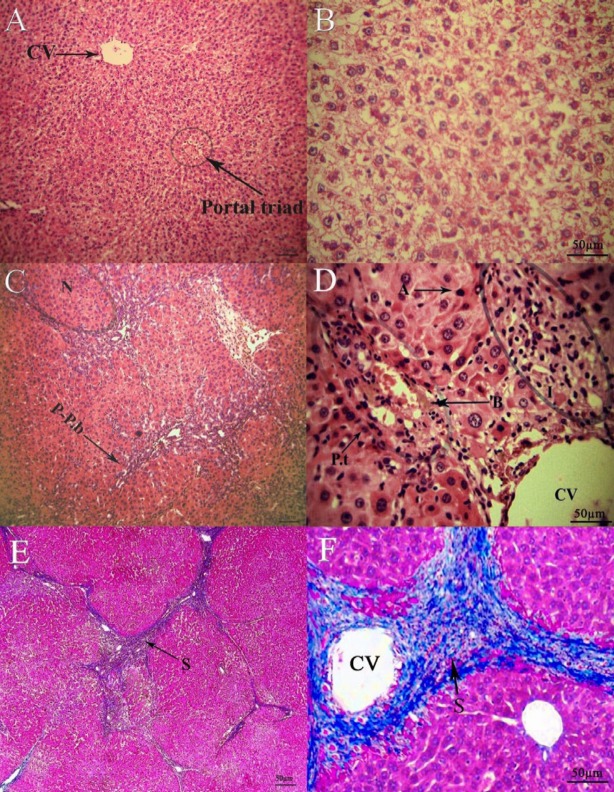
The relative SOCS1 mRNA expression in experimental groups; SOCS1 expression reduced in BDL rats than sham and increased in BDL/Thl rats than BDL (***P<0.001), mean±SEM. (One -way ANOVA, bonferroni post-test) (n=7)

BDL: Bile Duct Ligated; Thl: Thalidomide; SOCS1: Suppressor of Cytokine Signaling 1

We used RT-PCR to assess the expression of SOCS1 in rat liver and observed that a single band was visible in all of the study groups after ampliﬁcation of cDNA using a speciﬁc primer for SOCS1 (Figure 4). Our data showed that (Figure 5), relative SOCS1 expression decreased significantly in bile duct ligated group than the sham-operated group (*P*<0.001). Additionally, its expression was notable and significantly increase in BDL/Thl group compared with BDL/saline (*P*<0.001).

***Histopathology***

**Figure 6:**
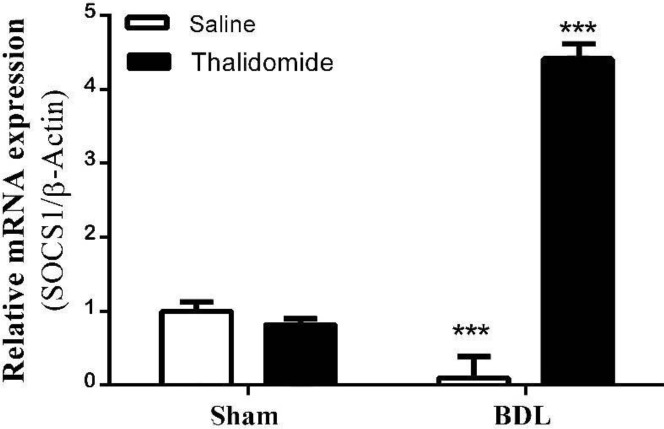
Hematoxylin-eosin staining of liver tissue; A: sham (100x), B: sham (200x), C: BDL (100x) N, micronodulation;P-P.b, portal-portal bridge; D: BDL(200x) P.t, portal triad; B, bilirubin pigments; A, apoptotic cells; I, lymphocytic infiltration. Masson-trichrome staining of liver tissue; E: BDL (100x), F: BDL (200x), S,fibrous septa

**Figure 7:**
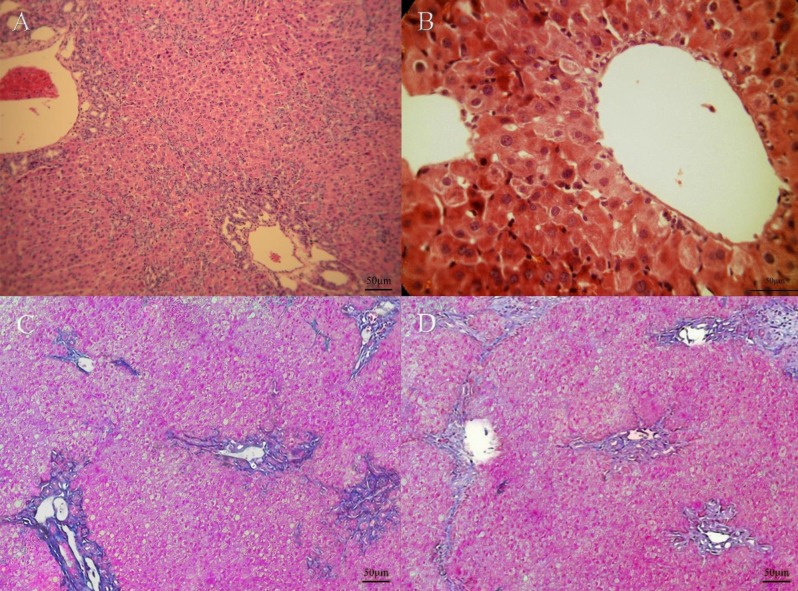
Hematoxylin-eosin staining of liver tissue; A: BDL/Thl (100x), B: BDL/Thl (200x). Masson-trichrome staining of liver tissue; C-D: BDL/Thl (100x). micronoulation,liver heterogeneity, bile ductule proliferation, collagen deposition and fibrotic septa bands decrease in cirrhotic rats by Thalidomide treatment

BDL: Bile Duct Ligated; Thl: Thalidomide

The hepatic lobules architecture in the Sham/Saline group was of high significance, and there was no ﬁbroplasia and inﬂammatory cell inﬁltration (Fig 6, A-B). Nevertheless, in the rats of BDL/ Saline group, the lobules of the liver were separated and encompassed by periportal and perivenular collagen deposition. Wide bridging ﬁbrosis (portal- portal and portal- central connection with ﬁbrotic bands) led to apparent pseudo-lobules and micronodules. Moreover, there was severity of hepatocytes that ultimately ends up in apoptosis and necrosis. Furthermore, a widespread inﬂammatory cell inﬁltration proliferating bile ductules (Fig 6C-F) was observed. Thalidomide treatment in bile duct ligated rats resulted in the improvement of necro-inflammation and lymphocytes infiltration. Additionally, thalidomide could shorten and disconnect septa in hepatic tissue that reduce the micronodular appearance and recover liver homogeneity (Fig 7A-D).

**Table 1:** The median and range of atrial maximum chronotropic response to β-adrenergic stimulation (R_max_) in study groups. (non-parametric Dunn’s test) (n=7)

Group	Rmax Median(Range)
Sham / Saline	156(145-195)
Sham / Thalidomide	152 (130-169)
BDL / Saline	137 (118-153)
BDL / Thalidomide	150 (138-154)

BDL: Bile duct ligated

**Table 2:** Hepatic and serum TNF-α, hepatic IL-6 and serum NO concentrations in experimental groups (a *P* <0.001, b *P*<0.01compared with sham/Saline) (c *P*<0.05 compared with BDL/Saline, mean±SEM. (One -way ANOVA, bonferroni post-test) (n=7)

Group	Hepatic TNF-α(Pg/gProtein)	Hepatic IL-6(Pg/gProtein)	Serum TNF-α(Pg/ml)	Serum NO(µM/L)
Sham/Saline	149.2±3.7	13.75±0.6	4.555±1.1	20.50±4.1
Sham/Thl	140.1±0.7	15.37±0.5	5.082±0.8	23.99±2.3
BDL/Saline	392.6±0.9a	22.05±1.9 b	36.91±1.1a	54.92±2.864b
BDL/Thl	352.6±1.5c	19.24±0.9c	29.60±1.7c	31.35±1.374c

SOCS1: Suppressor of Cytokine Signaling 1

BDL: Bile duct ligated

## Discussion

Cirrhotic cardiomyopathy is a complication of cirrhosis wherein physical or pharmacological stimulation cannot induce an adequate increase in heart rate (2). This condition is related to the development of a hemodynamic disorder which is called “hyperdynamic circulatory syndrome” (HC), and characterizes both portal hypertension (PTH) and NO overproduction (3). Moreover, it was shown that TNF-α and IL-6 levels are elevated in cirrhosis, and these enhancements are associated with HC intensification (4, 5, 21). Furthermore, the expression of SOCS1 as a modulator of hepatic inflammation decreases in cirrhosis condition (11). As an immunomodulatory and anti-inflammatory drug, thalidomide decreases the TNF-α and IL-6 levels (22). Although previous studies have indicated that thalidomide inhibits fibrosis and inflammation progression in cirrhosis, there are not enough studies concerning its role in cirrhotic cardiomyopathy and also the involvement of SOCS1 in this effect. Hence, in the present study we examined the effect of chronic thalidomide administration in BDL rats on atrial chronotropic hyporesponsiveness to β-adrenergic stimulation. Our results revealed that thalidomide could improve atrial response to β-adrenergic stimulation in cirrhotic rats. The histopathology finding of the present report indicated that bile duct proliferation, collagen deposition, and inflammatory cell infiltration could increase in the liver after cirrhosis. Moreover, this research indicated that thalidomide treatment could improve the histopathological conditions of cirrhotic liver. This is consistent with the findings presented by Napoli *et al*. in their investigations on 28-day BDL rats (23). The present results revealed that TNF-α and IL-6 levels could increase in cirrhotic rat livers compared to sham-operated animals, and chronic thalidomide administration could significantly diminish the abovementioned cytokine in the BDL group. It was previously indicated that the elevated levels of hepatic TNF-α and IL-6 in BDL rats were decreased by thalidomide administration in BDL animals (24). Following the ligation of the common bile duct, the hepatic stellate cell is activated by TNF-α and IL-6. These cytokines caused collagen deposition, fibrotic septa band formation, and the deterioration of microvasculature (25, 26). Hence, thalidomide is possibly able to improve liver function and histopathological conditions in BDL rats. However, further studies are required for more clarification. Additionally, the findings of the present study indicated that NO and TNF-α levels in serum could be augmented in the BDL group compared to the sham-operated one. Plasma-elevated NO was well demonstrated by Mani *et al*. in BDL rats (27), and plasma raised TNF-α concentration was adequately indicated by Fernandez-Martınez *et al*. in the BDL rats (1). Furthermore, it was exhibited that NO overproduction could be mediated by TNF-α (28). In accordance with previous studies, we showed that portal vein pressure (PVP) could significantly rise along with the establishment of HC in BDL rats (18, 29). The findings of the present study indicated that SOCS1 mRNA expression was lower in bile duct ligated rats compared to sham-operated ones. This phenomenon was reported by Yoshida *et al*. in the case of dimethylnitrosamine-induced cirrhosis in mice (11). It was determined that the deficiency of SOCS1 could induce severe hepatic disorders (8, 10, 30). It has also been shown that SOCS1 gene is silenced by hypermethylation in cirrhosis (31, 32). The chronic administration of thalidomide in this study could ameliorate collagen deposition, inflammatory cell infiltration, and an overall liver uniformity. In agreement with the results, Ta-Sen Yeh *et al*. indicated that thalidomide could inhibit hepatic necroinﬂammation in cirrhotic rats (33). Therewith, thalidomide reduced hepatic TNF-α and IL-6 levels in cirrhotic rats, and reversed amplified systemic NO and PTH in those rats. Consistent with this finding, Ying-Ying Yang *et al*. reported that thalidomide could diminish the hepatic levels of TNF-α, IL-6 and increase PTH, in cirrhotic rats (33). Moreover, it was previously displayed that excessive NO and PVP could be reduced by thalidomide therapy in the model of portal hypertension. It seems that all of the above-mentioned actions of thalidomide were mediated by TNF-α inhibition. Furthermore, the inhibition of IL-6 by thalidomide is partly responsible for the reduction of collagen deposition and fibrosis. Surprisingly, this study is the first research indicating that thalidomide could significantly increase SOCS1 mRNA levels in cirrhotic rats. It was shown that thalidomide analog could upregulate the SOCS1 expression through the demethylation of its silenced promoter region (34). Our results revealed that with chronic common bile duct ligation, the responsiveness of isolated atria chronotropic to β-adrenergic stimulation, is impaired. This finding was clearly noted by Mani *et al*. and Jazaeri *et al*. concerning BDL rats (27, 35). By HC progression and effective vasodilation, a systemic hypotension occurs. To compensate, tachycardia and cardiac overstrain take place that result in insufficient myocardium response to stress in long term. On the basis of our results, the chronic thalidomide administration in cirrhotic rats removes the hyporesponsiveness of isolated atria to the chronotropic effect of isoproterenol stimulation.

## Conclusion

The possible mechanism of action of thalidomide for the improvement of cardiac function in cirrhotic rats is mediated by the amelioration of disturbed liver architecture, the decrease of IHR, PTH, and the augmentation of NO systemic in cirrhosis.

## Conflicts of Interest

None of the authors of this paper has a financial or personal relationship with other people or organizations that could inappropriately influence or bias the content of the paper.
